# Enhancing the anti-glioma therapy of doxorubicin by honokiol with biodegradable self-assembling micelles through multiple evaluations

**DOI:** 10.1038/srep43501

**Published:** 2017-02-27

**Authors:** Xiang Gao, Ting Yu, Guangya Xu, Gang Guo, Xiaoxiao Liu, Xin Hu, Xiang Wang, Yanhui Liu, Qing Mao, Chao You, Liangxue Zhou

**Affiliations:** 1 Department of Neurosurgery, State Key Laboratory of Biotherapy, West China Hospital, Sichuan University and Collaborative Innovation center, Chengdu, 610041, PR China; 2 Department of Pharmacology, Yale School of Medicine, Yale University, New Haven, Connecticut, 06520, USA; 3 Institute of Neurosurgery, West China Hospital, West China Medical School, Sichuan University, Chengdu, 610041, PR China

## Abstract

Combination chemotherapy is an important protocol in glioma therapy and honokiol shows synergistic anticancer effects with doxorubicin. In this paper, honokiol (HK) and doxorubicin (Dox) co-loaded Methoxy poly(ethylene glycol)-poly(ε-caprolactone) (MPEG-PCL) nanoparticles were prepared with a assembly method. The particle size (about 34 nm), morphology, X-ray Powder Diffraction (XRD), *in vitro* release profile, cytotoxicity and cell proliferation effects were studied in detail. The results indicated that honokiol and doxorubicin could be efficiently loaded into MPEG-PCL nanoparticles simultaneously, and could be released from the micelles in an extended period *in vitro*. In addition, honokiol and doxorubicin loaded in MPEG-PCL nanoparticles could efficiently suppress glioma cell proliferation and induce cell apoptosis *in vitro*. Furthermore, Dox-HK-MPEG-PCL micelles inhibited glioma growth more significantly than Dox-MPEG-PCL and HK-MPEG-PCL in both nude mice and zebrafish tumor models. Immunohistochemical analysis indicated that DOX-HK-MPEG-PCL micelles improved Dox’s anti-tumor effect by enhancing tumor cell apoptosis, suppressing tumor cell proliferation, and inhibiting angiogenesis. Our data suggest that Dox-HK-MPEG-PCL micelles have the potential to be applied clinically in glioma therapy.

Malignant gliomas account for approximately 70% of the 22850 new cases of malignant primary brain tumor diagnosed in adults in the United States each year^1^,^2^. Glioma, with a survival time of 12–15 months and a 5-year survival rate of less than 5%, has been regarded as one of the most lethal and aggressive types of brain tumors because of its insusceptibility to conventional therapies^3^,^4^. Chemotherapy is the most common method to treat glioma; however, due to the physical obstacle formed by the blood-brain barrier (BBB), which obstructs the drug accumulation and uptake in gliomas, the efficiency of chemotherapy is greatly reduced, and the side effects are severe^5^. Hence, effective strategies are urgently demanded for the treatment of glioma. As a promising strategy, the combination of nanotechnology and conventional chemotherapy brings hope for the treatment of glioma^6^,^7^.

Honokiol (HK: Fig. 1A) is a bio-active component of the bark of the Chinese traditional herb *Magnolia officinals*^8^. Previous studies have demonstrated the variety of pharmacological effects of HK, including anti-oxidative, anti-depressant, and anti-inflammatory effects^9^,^10^,^11^. In recent decades, many studies have shown that HK possesses extensive anticancer efficacy^12^. HK can inhibit angiogenesis, tumor invasion, migration, and growth both *in vivo* and in multiple cancer cell lines^13^,^14^,^15^. Cellular studies provide novel insights into the underlying anticancer mechanisms of HK. For example, the biological effects of HK inhibit the phosphorylation of ERK, Akt and c-Src^16^. HK targets the signal transducer and activator of transcription 3/Zeb1/E-cadherin axis to effectively inhibit EMT (epithelial-mesenchymal transition) in breast cancer^17^. In addition, HK showed synergistic antitumor effects when combined with chemotherapy^18^. DOX (Fig. 1B) as a strong candidate for chemotherapy of the central nervous system (CNS), has been shown to arrest cell growth and induce apoptosis in malignant glioma cell lines. The survival of glioma patients can be improved by direct intratumoral infusion of DOX. However, the clinical application of DOX is inevitably limited by its serious side effects, including gastrointestinal toxicity, myelosupression, and cardiotoxicity^19^. Furthermore, the wider application of HK is also limited due to its high hydrophobicity. Therefore, it is critical to develop a novel formulation for the co-delivery of HK and DOX.

Nanotechnology has played an important role in drug delivery systems (DDS)^20^,^21^. Nanomaterials, such as nanoparticles, liposomes, and polymeric micelles, have been increasingly applied as carriers in the delivery of hydrophobic drugs^22^,^23^,^24^. Micelles can be prepared from the biodegradable, amphiphilic copolymers of poly (ε-caprolactone) (PCL) and polyethylene glycol (PEG), which provide the hydrophobic and hydrophilic components, respectively^25^. The hydrophobic PCL segments pack together to form the core which serves as a potential nanocontainer of hydrophobic drugs, while the outer hydrophilic PEG region serves as a stabilizing shell; thus, the encapsulation of hydrophobic drugs into the core-shell nanostructure can render them completely dispersible in solution^26^. As such, these micelles provide an attractive method to make nanovector-based formulations for hydrophobic drugs. In this study, PEG-PCL micelles were used to encapsulate HK and DOX, creating DOX-HK-M nano-micelles. The effect of DOX-HK-M nano-micelles on glioma was then evaluated. Our results suggest that DOX-HK-M nano-micelles provide a novel formulation of HK and DOX, with promising applications in glioma therapy.

## Results

### Preparation and characterization of DOX-HK-M nano-micelles

**Figure 1:**
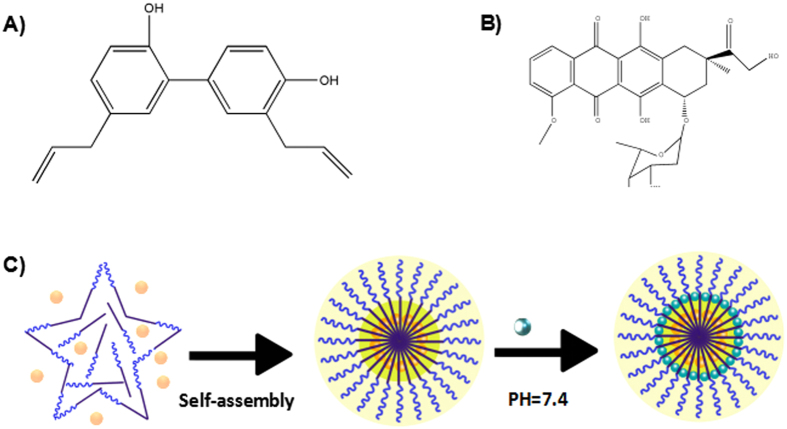
Preparation of DOX-HK-M nano-micelles. The HK-Dox-M nano-micelles were prepared in two-steps by a self-assembly method. First the HK (**A**) and MPEG-PCL (**B**) were co-dissolved in acetone, and the mixture was then added into water with stirring. Secondly, 1 mL of 10x phosphate-buffered saline (PBS: 0.1 M, pH 7.4) was added to HK/MPEG-PCL nano-micelles (8 mL) and mixed completely. Next, 1 mL of doxorubicin solution (5 mg/mL) was dripped slowly into the mixture under continuous stirring by a mechanical stirrer.

DOX-HK-M nano-micelles were prepared by a two-step self-assembly procedure, as shown schematically in Fig. 1C. First, MPEG-PCL copolymer and HK were co-dissolved in acetone. The organic phase was then evaporated in a rotary evaporator under reduced pressure, and subsequent addition of water formed the core-shell structured HK/MPEG-PCL nano-micelles with core-encapsulated HK. Afterward, phosphate-buffered saline (PBS) and doxorubicin solution were added into the HK/MPEG-PCL nano-micelles under continuous mechanical stirring, thus completing the DOX-HK-M nano-micelle preparation. This self-assembly procedure of amphiphilic MPEG-PCL, HK, and DOX created core-shell structured DOX-HK-M nano-micelles with core-encapsulated HK and DOX.

**Figure 2:**
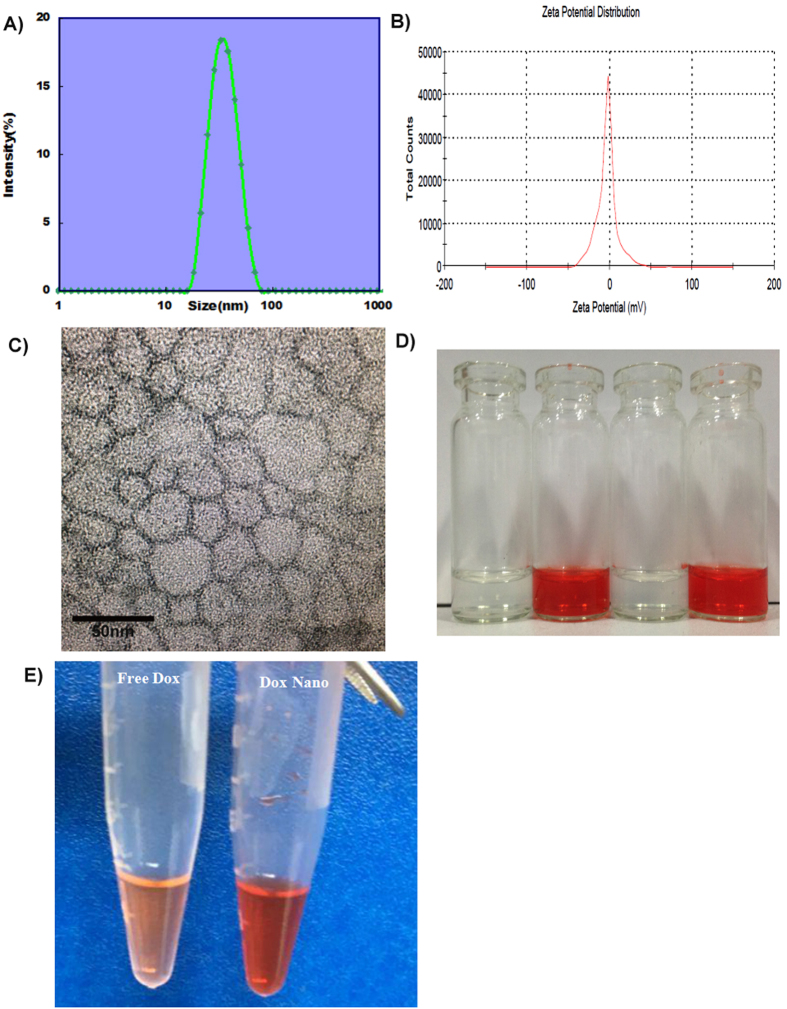
Characterization of Dox-HK-M nano-micelles. (**A**) Size distribution of Dox-HK-M nano-micelles; (**B**) zeta potential distribution of Dox-HK-M; (**C**) TEM image of Dox-HK-M; (**D**) photos of empty MPEG-PCL micelles, Dox-M micelles, HK-M micelles, and Dox-HK-M micelles in PBS solution (from left to right, respectively). (**E**) Free DOX and DOX-HK-M were filtered through ultrafiltration tube.

The DOX-HK-M nano-micelles were characterized in detail. The drug loading (DL) and Encapsulation efficiency (EE) of Dox and HK in DOX-HK-M nano-micelles were 5% and 93.4% (for Dox), and 5% and 99.8% (for HK). The particle size distribution spectrum of freshly-prepared DOX-HK-M nano-micelles is shown in Fig. 2A. The polydispersity index (PDI) of the prepared DOX-HK-M nano-micelles was 0.12, with a mean particle size of 34 nm, indicating the narrow particle size distribution of DOX-HK-M nano-micelles. The zeta potential of DOX-HK-M nano-micelles was −2.3 mv, presented in Fig. 2B. Furthermore, the morphology of DOX-HK-M nano-micelles was determined by transmission electron microscopy (TEM), which is shown in Fig. 2C. The TEM images revealed that the DOX-HK-M nano-micelles were spherically-shaped in aqueous solution with a mean diameter of 29 nm, which was in good accordance with the results of dynamic light scattering (DLS). DLS allows for observation of the diameter of particles in aqueous solution, whereas TEM provides the diameter of particles in dry powder. The structure of amphiphilic block polymeric micelles was looser in aqueous solution, which could explain the slightly larger particle size determined by DLS than that by TEM. Combination of the above results showed that the prepared DOX-HK-M nano-micelles were stable and homogeneous in aqueous solution.

The appearance of DOX-HK-M nano-micelles in aqueous solution is shown in Fig. 2D. HK couldn’t be dissolved in aqueous solution, instead forming a turbid white slurry, while the DOX-HK-M nano-micelles could be well-dissolved in aqueous solution as demonstrated by the clear solution. What is more, the dox can be filtered from ultrafiltration tube in the free DOX group and not be filtered in the DOX-HK-M group (shown in Fig. 2E). Those indicated that the HK and DOX were incorporated into the micelles.

### X-ray Powder Diffraction

**Figure 3:**
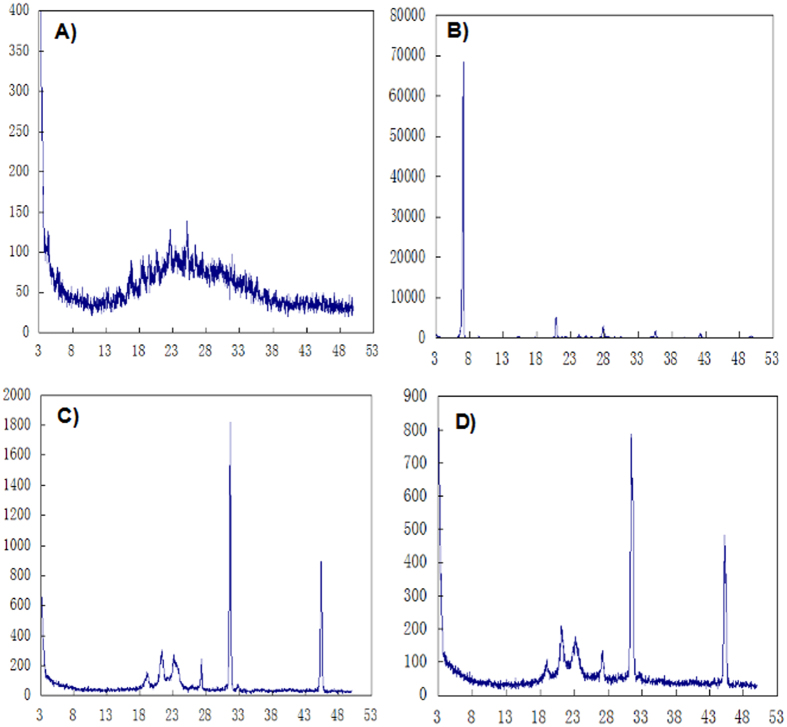
XRD analysis. Crystallographic assays of Dox (**A**), HK (**B**), blank MEPG-PCL (**C**), and Dox-HK-M micelles (**D**) were using an X-ray diffractometer.

X-ray Powder Diffraction (XRD) spectra of the HK powder, DOX powder, lyophilized blank MPEG-PCL nano-micelles, and DOX-HK-M nano-micelles are presented in Fig. 3. Compared with the XRD diagram of Dox and HK powder, the specific X-ray diffraction peaks of HK and Dox power disappeared in the diagram of DOX-HK-M nano-micelles.

### *In vitro* drug release behavior

**Figure 4:**
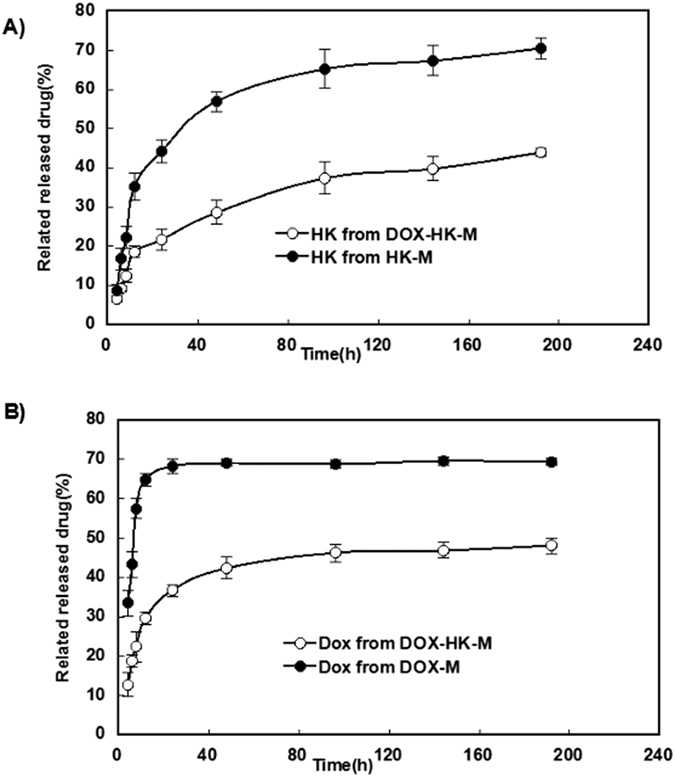
Release study *in vitro*. Release profile of Honokiol or doxorubicin micelles *in vitro* was studied using a dialysis protocol. Honokiol was released from HK/MPEG-PCL or Dox-HK-M (**A**) and doxorubicin was released from Dox/MPEG-PCL or Dox-HK-M (**B**).

The release profiles of HK-M nano-micelles, DOX-M nano-micelles and DOX-HK-M nano-micelles in PBS (pH 7.4, 37 °C) with 10% fetal bovine serum are presented in Fig. 4. Dox and HK were able to be released from the DOX-HK-M nano-micelles, and the cumulative release rates of HK or DOX from the DOX-HK-M nano-micelles were slower when compared with the HK-M nano-micelles or DOX-M nano-micelles alone.

### *In vitro* cytotoxicity evaluation

**Figure 5:**
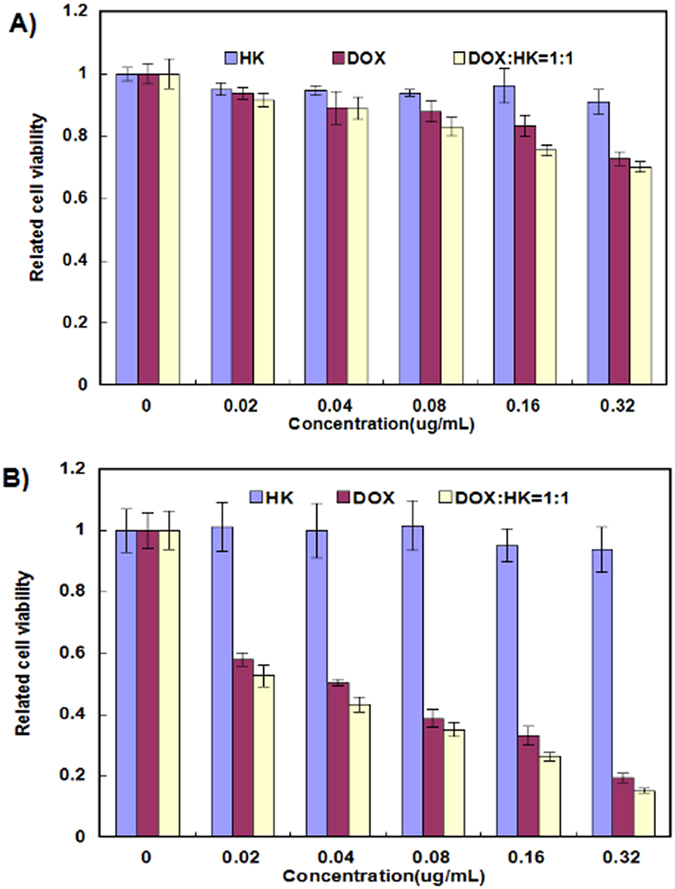
The MTT assay. The MTT assay (Mean ± SEM, n = 6) showed that the combination group (ratio of Honokiol to doxorubicin, 1:1) was more effective than HK and Dox alone at concentrations of 0.02 μg/mL, 0.04 μg/mL, 0.08 μg/mL, 0.16 μg/mL, and 0.32 μg/mL at 24 h (**A**) or 48 h (**B**).

The *in vitro* cytotoxicity profiles of HK-M nano-micelles, DOX-M nano-micelles, and DOX-HK-M nano-micelles were determined by an MTT assay using C6 glioma cells for 24 (Fig. 5A) and 48 hours (Fig. 5B). As shown in Fig. 5, both DOX-M nano-micelles and DOX-HK-M nano-micelles—but not HK-M nano-micelles—inhibited the cell viability of C6 glioma cells in a dose-dependent manner. Compared with the HK-M nano-micelles and Dox-M nano-micelles, the DOX-HK-M nano-micelles conferred greater cytotoxicity to C6 glioma cells under the same dose of HK or DOX.

### Induction of apoptosis by DOX-HK-M nano-micelles

**Figure 6:**
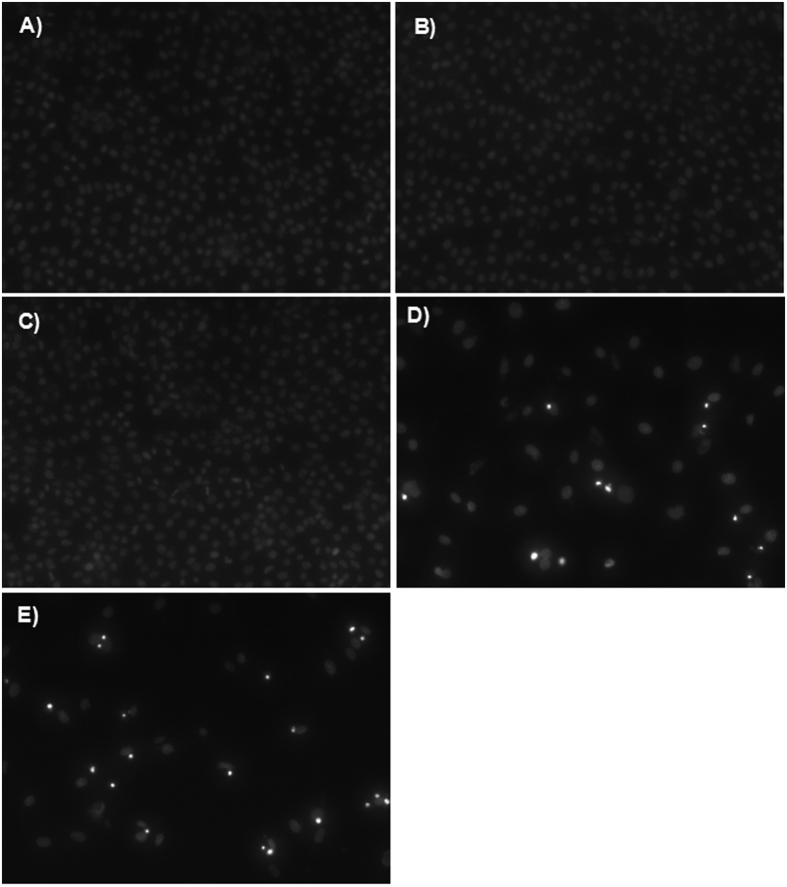
Hoechst staining. C6 cells were treated with NS (**A**), empty MPEG-PCL (**B**), HK-M (**C**), Dox-M (**D**), or HK-Dox-M (**E**) at drug concentrations of 80 ng/mL for 48 h, and then stained with Hoechst 33342 for fluorescence microscopy observations. And this test was repeated for three times.

Hoescht staining analysis was performed on C6 glioma cells in order to investigate whether apoptosis contributed to the inhibition of cell growth. Stains were performed on C6 glioma cells in order to investigate the apoptotic potential of HK-M, DOX-M, and DOX-HK-M nano-micelles. As seen in Fig. 6, images showed more apoptotic cells with condensed and fragmented nuclei in DOX-HK-M nano-micelles group was than those of other groups.

**Figure 7:**
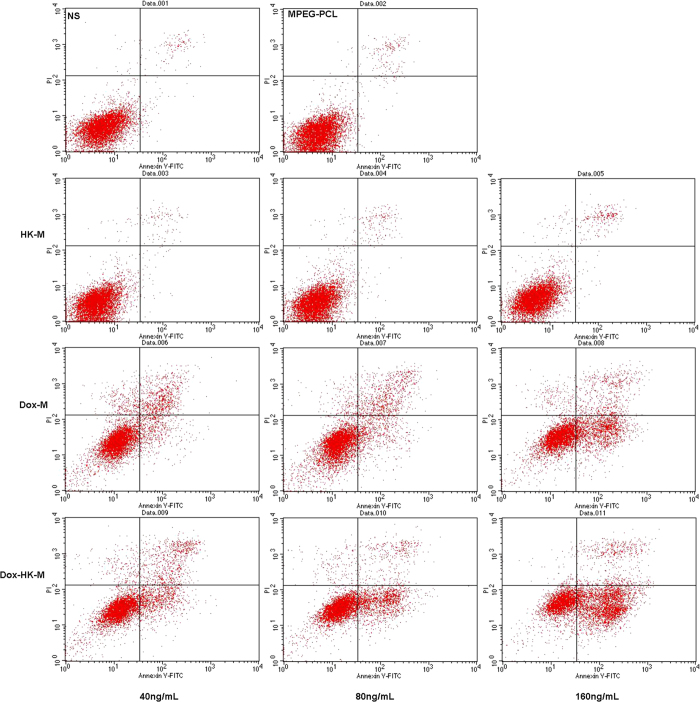
Cell apoptosis assay with Flow cytometry. C6 glioma cancer cells were treated with HK-M, Dox-M, or HK-Dox-M at concentrations ranging from 40 ng/mL to 160 ng/mL for 48 h. Afterward, the cells were stained with PI and Annexin V for FCM assay. And this test was repeated for three times.

Moreover, Flow cytometry (FCM) analysis of PI/Annexin V staining was used to investigate the apoptotic potential of DOX-M nano-micelles, including both early apoptotic (Annexin V^+^/PI^−^) and late apoptotic (Annexin V^+^/PI^+^) cells. As shown in Fig. 7, the percentage of apoptosis in DOX-HK-M nano-micelles (25.77%, 28.71%, 54.95%) was significantly higher than those in NS (2.76%), blank micelles (3.38%), HK-M nano-micelles (2.31%, 2.67%, 3.3%) and DOX-M nano-micelles (20.04%, 22.88%, 34.87%). The consistent results of Hoescht staining and FCM analysis suggested that the DOX-HK-M nano-micelles could induce more cells to enter apoptosis than that of HK-M nano-micelles or DOX-M nano-micelles.

### Cellular uptake of DOX-HK-M nano-micelles

**Figure 8:**
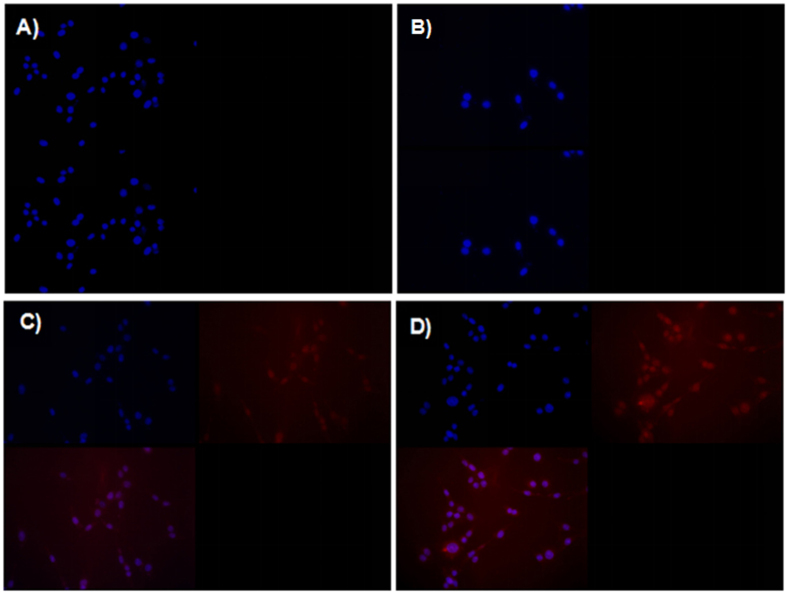
Enhancement of cell uptake drug with fluorescence microscopy observation. C6 glioma cancer cells were treated with EM (**A**), HK-M (**B**), Dox-M (**C**), or HK-Dox-M (**D**) at concentrations of 80 ng/mL for 4 h, at which point the cells were stained with Hoechst 33342 for fluorescence microscopy observations. And this test was repeated for three times.

**Figure 9:**
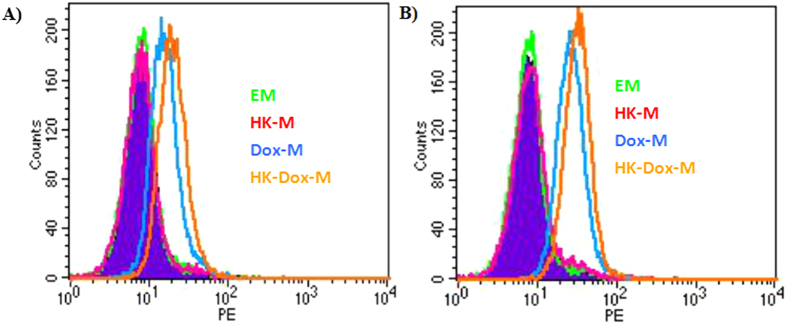
Cell uptake as seen via the FCM test. C6 glioma cancer cells were treated with EM, HK-M, Dox-M, or HK-Dox-M at concentrations of 40 ng/mL (**A**) or 80 ng/mL (**B**) for 4 h, at which point the cells were collected for FCM test. And this test was repeated for three times.

To reveal the mechanism of DOX-HK-M nano-micelles in enhancing cellular cytotoxicity and inducing apoptosis, a cellular uptake study of the nano-micelles was performed on C6 glioma cells. As shown in Fig. 8, no red fluorescence could be observed in the blank-M nano-micelles group, nor the HK-M nano-micelles group, at 4 hours. Compared with the DOX-M nano-micelles group, the DOX-HK-M nano-micelles group exhibited a brighter red fluorescence at 4 hours, indicating increased cellular uptake of DOX; the same result was also determined by FCM analysis. As seen in Fig. 9, the FCM histograms elucidated that the cellular uptake of DOX in DOX-HK-M nano-micelles group was greater than that of the other groups after 4 h incubation.

### DOX-HK-M nano-micelles inhibited embryonic angiogenesis in a Tg (FLK-1: EGFP) zebrafish model

**Figure 10:**
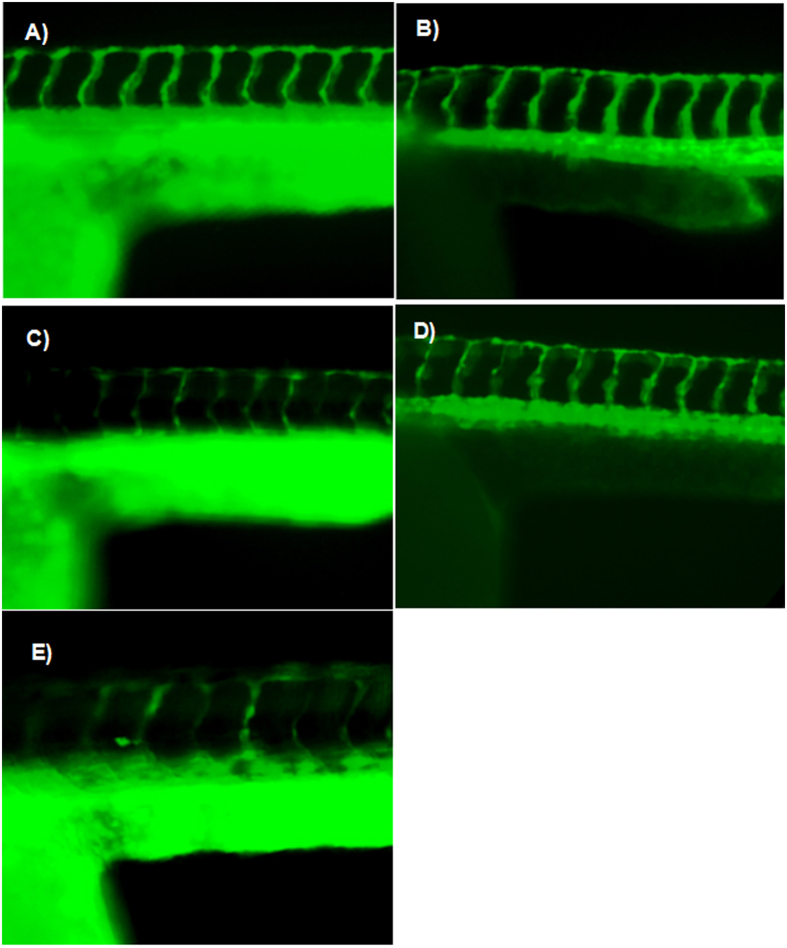
Anti-angiogenic effects of HK-Dox/MPEG-PCL. Normal embryos (**A**), embryos treated with empty MPEG-PCL (**B**), embryos treated with HK/MPEG-PCL micelles (**C**), embryos treated with Dox/MPEG-PCL micelles (**D**), and embryos treated with HK-Dox/MPEG-PCL micelles (**E**). Embryos treated with HK-Dox/MPEG-PCL micelles showed defective vascular formation of variable severity, with intersegmental vessels either sprouting abnormally or failing to form in comparison with control embryos.

The anti-angiogenesis effects of DOX-HK-M nano-micelles were investigated using transgenic zebrafish model embryos. Zebrafish embryos at 14 hour post fertilization (hpf)were incubated with Holtfreter’s solution (control), blank micelles, H-M nano-micelles, DOX-M nano-micelles and DOX-H-M nano-micelles for 10 h. At 24 hpf, the zebrafish embryos were anaesthetized with 0.01% tricaine, and followed by taking images of blood vessels by a fluorescence microscope. Shown in Fig. 10, DOX-HK-M nano-micelles dramatically inhibited the growth of intersegmental vessels (ISVs) compared with other groups. The lengths of ISVs in DOX-H-M nano-micelles and HK-M groups were significantly shorter than those in the control, blank micelles, HK-M, and DOX-M nano-micelles groups.

### *In vivo* antitumor effects in tumor xenograft zebrafish models

**Figure 11:**
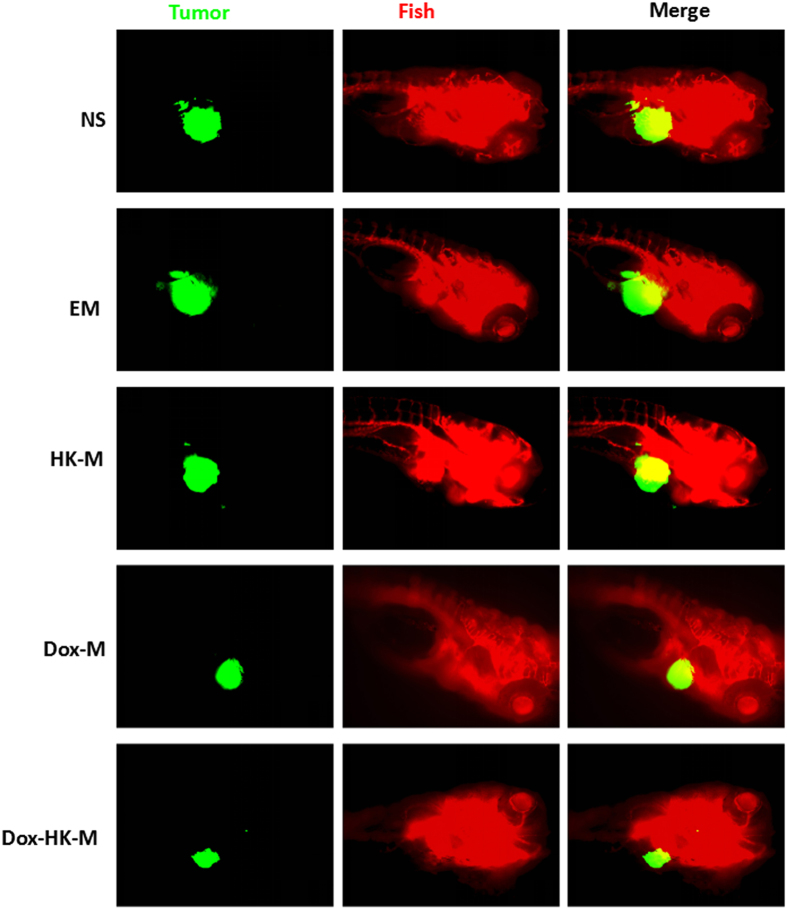
Antitumor effects of Dox-HK/MPEG-PCL in zebrafish tumor models. Zebrafish were injected with 300 tumor cells into the perivitelline space using a Cell Tram Vario injector with a glass micropipette. At 24 h post-implantation, Tumors from zebrafish treated with NS, empty micelles (EM), HK-M, Dox-M, or Dox-HK-M micelles are shown. The Dox-HK/MPEG-PCL micelles inhibited tumor growth in zebrafish more effectively than other groups.

Three days after injection of perivitelline into the zebrafish, U87 cells formed a solid tumor (green fluorescence, Fig. 11). Shown in Fig. 11, antitumor effects were observed in HK-M, DOX-M, and DOX-H-M nano-micelles groups when compared with the control and blank micelles group. As detected by confocal microscopy, the tumors in the DOX-H-M nano-micelles group were the smallest of all the groups, including the H-M and DOX-M nano-micelles groups; no antitumor effects was observed in blank micelles group. The average tumor volume in the DOX-HK-M nano-micelles group was significantly smaller than that of control, blank micelles, HK-M nano-micelles, and DOX-M nano-micelles groups. These data indicated that HK could enhance the anti-glioma cancer activity of Dox *in vivo*.

### *In vivo* antitumor effects in a subcutaneous tumor model

**Figure 12:**
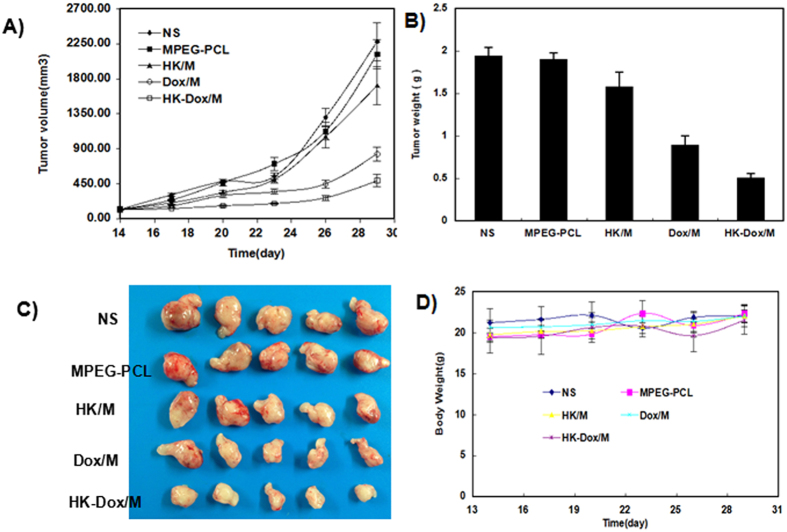
The anti-glioma effect of HK-Dox-M *in vivo* inhibits the growth of established C6 tumors in nude BALB/c mice. (**A**) Tumor volume was measured every 3 days. (**B**) Weights of the tumors. (**C**) Representative photos of the tumors in each treatment group. (**D**) The body weights of the mice were measured every three days. This indicated that HK-Dox-M could inhibit glioma growth *in vivo* more effectively than other groups. (Mean ± SEM, n = 5).

Subcutaneous injection of C6 glioma cells was used to evaluate the antitumor effects of DOX-HK-M nano-micelles. Figure 12A and B show the tumor growth curves and weight in each group, respectively; Fig. 12C shows representative tumors from each group. According to the results, DOX-HK-M nano-micelles exhibited stronger antitumor effects than that of the other groups, while the blank micelles exhibited no antitumor effect at all. Figure 12D shows the body weight curves of different groups, demonstrating that there was no change among the groups.

### Determination of tumor cell apoptosis

**Figure 13:**
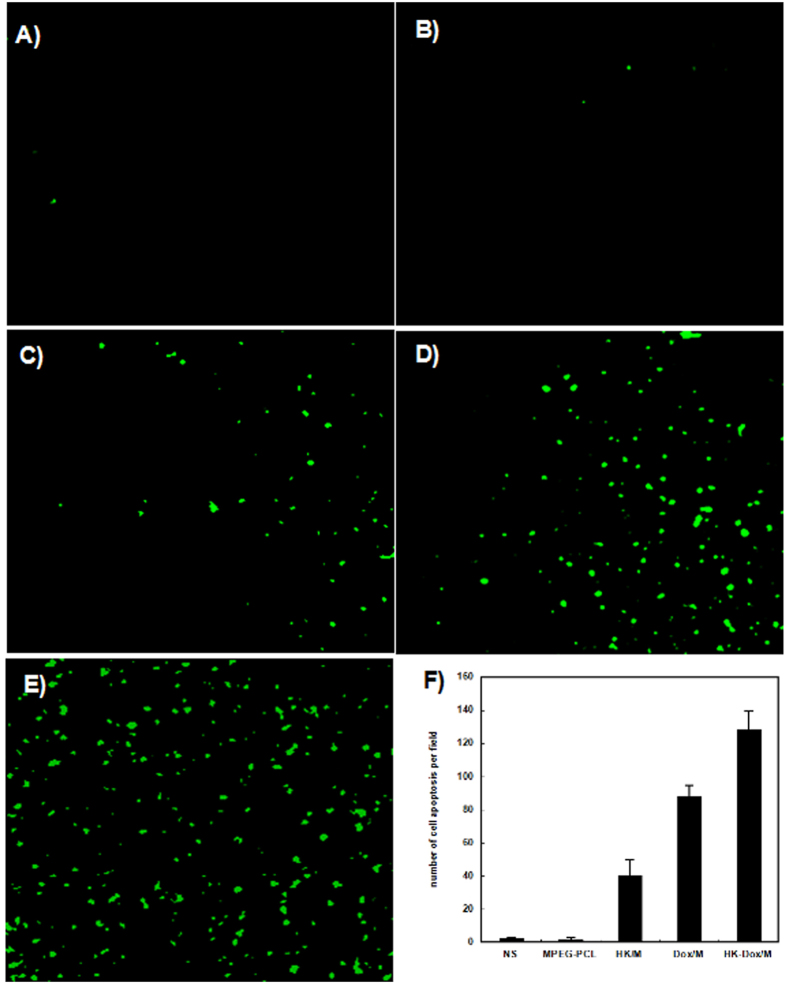
The TUNEL assay. Tumor tissue was collected after the final treatment and tumor sections of NS (**A**), blank micelles (**B**), HK-M (**C**), Dox-M (**D**), or Dox-HK-M (**E**) were stained with TUNEL for the cell apoptosis assay. (**F**) The amount of cell apoptosis in each group. (Mean ± SEM, n = 5).

An immunofluorescent TUNEL assay was used to evaluate the degree of apoptosis in each group; only in the regions of intact tumor cells were the TUNEL positive cells counted, while the cells in the central necrotic region were excluded. As shown in Fig. 13A–E, there were more apoptotic tumor cells in the DOX-HK-M nano-micelles group than in other groups. In Fig. 13F, the apoptotic index in the DOX-HK-M nano-micelles group (128 per field) was significantly higher than that in control (2 per field, p < 0.05), blank micelles (2 per field, p < 0.05), HK-M nano-micelles (41 per field, p < 0.05) and DOX-M nano-micelles (88 per field, p < 0.05) groups, while there is no difference between the control and blank micelles groups.

### Assessment of Microvessel density (MVD)

**Figure 14:**
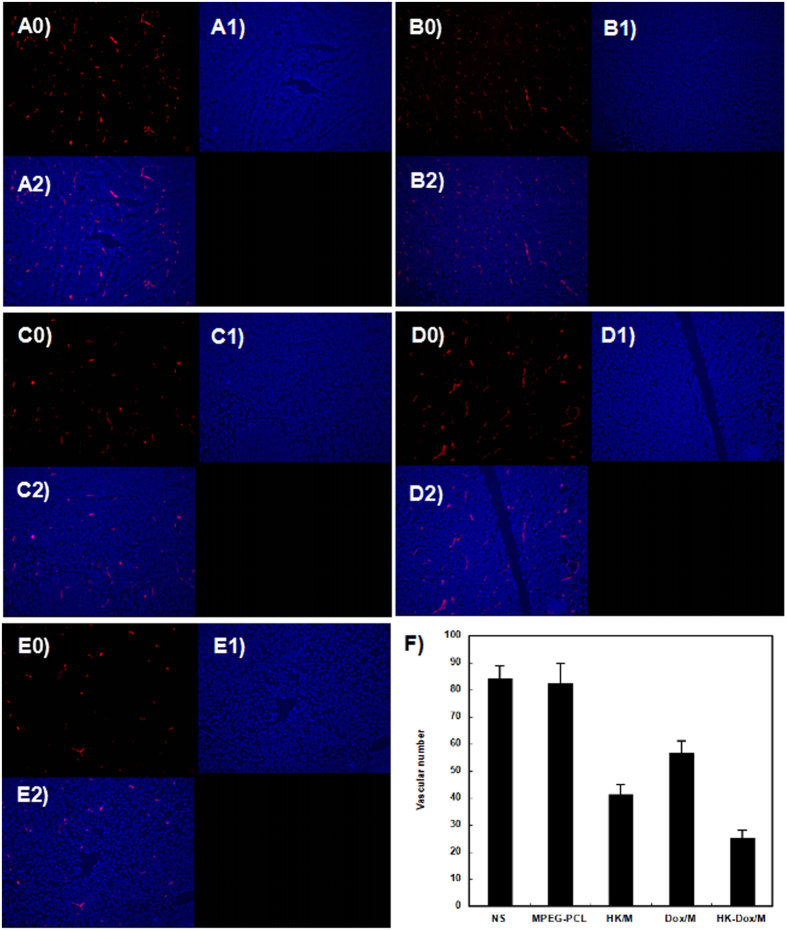
Tumor sections of each treatment group were stained with CD31 for evaluating the microvessel density (MVD). Tumor tissue was collected after the final treatment and tumor sections of NS (**A**), empty micelles (**B**), HK-M (**C**), Dox-M micelles (**D**), and Dox-HK-M (**E**) were stained with CD31 antibody. The CD31 assay vascular number (Mean ± SEM, n = 5) (**F**). The Dox-HK-M treatment caused inhibition of angiogenesis in the tumors. This implies that anti-angiogenic effects may be a mechanism of inhibiting C6 cancer by HK-M and Dox-HK-M *in vivo*, and that Dox-HK nano micelles inhibited more angiogenesis than other groups.

Immunofluorescent staining of CD31 in tumors was employed to quantitatively evaluate the anti-angiogenic activity of DOX-HK-M nano-micelles by assessing MVD. As shown in Fig. 14A–E, fewer microvessels were observed in tumor tissue of the DOX-HK-M nano-micelles group compared to that of other groups. The MVD in tumor tissue of the DOX-HK-M nano-micelles group (25 per field) was significantly lower than that of the control (84 per field, p < 0.05), blank micelles (83 per field, p < 0.05), HK-M nano-micelles (42 per field, p < 0.05), and DOX-M nano-micelles (56 per field, p < 0.05) groups.

### Alginate-encapsulated tumor cell assay

**Figure 15:**
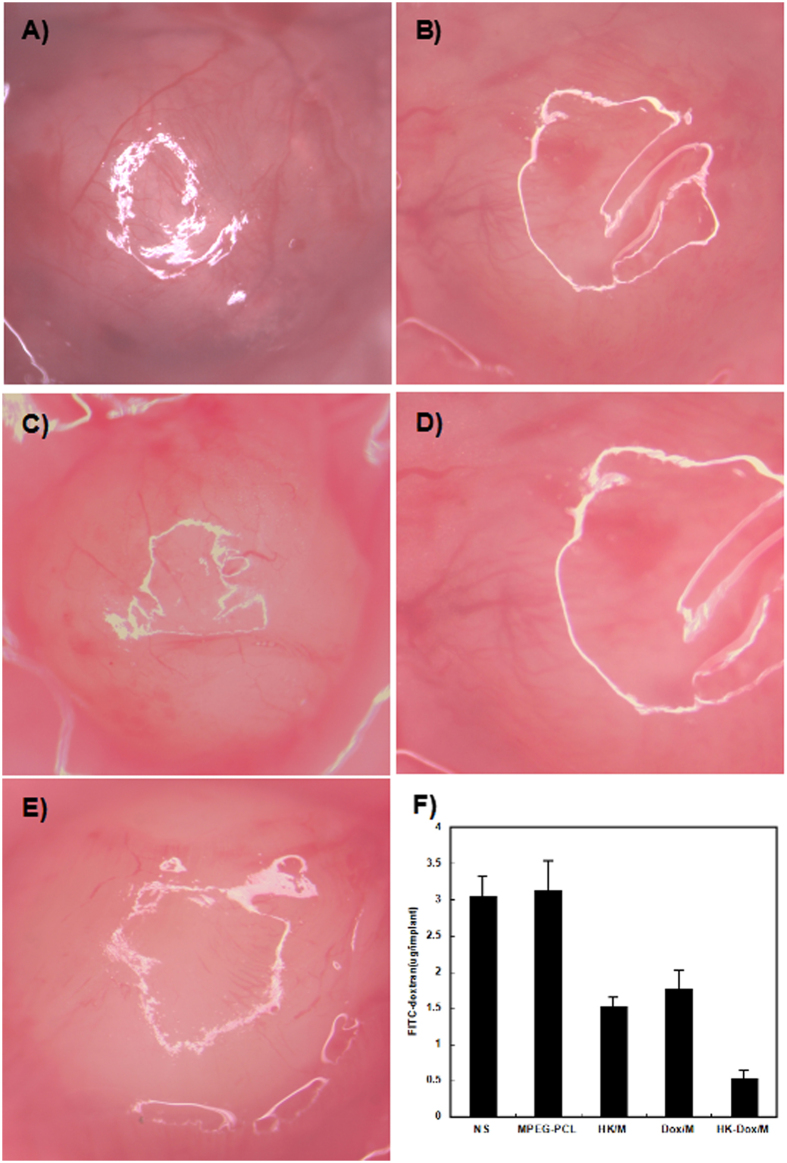
Alginate wrapping tumor cell inducing angiogenesis assay. C6 glioma cells were re-suspended in an alginate solution and then dropped into a calcium chloride solution to form alginate beads containing tumor cells. At day 0, the prepared alginate beads were subcutaneously implanted into an incision made on the dorsal sides of nude BALB/c mice. Afterwards, fifteen bead-bearing mice were randomly assigned to five groups (3 mice per group), and were intravenously injected with NS (control), blank micelles (3 mg/kg), HK-M nano-micelles (3 mg/kg), DOX-M nano-micelles (3 mg/kg), or DOX-HK-M nano-micelles (3 mg/kg) once a day for 3 days. Finally, mice were intravenously injected with 0.1 mL of a 100 mg kg-1 FITC-dextran solution at day 12. Representative images of alginate beads in NS (**A**), blank micelles (**B**), HK-M (**C**), Dox-M (**D**), Dox-HK-M (**E**), and uptake of FITC–dextran in each group (**F**) (Mean ± SEM, n = 5).

An alginate-encapsulated tumor cell assay was used to further investigate the anti-angiogenic effects of DOX-HK-M nano-micelles *in vivo*. As seen in Fig. 15A–D, sparser blood vessels in alginate beads were observed in the DOX-HK-M nano-micelles group than that of other groups. Uptake of FITC-dextran was also measured in order to quantify angiogenesis in the alginate implant. As shown in Fig. 15E, uptake of FITC-dextran in the DOX-HK-M nano-micelles group (0.53 μg per bead) was significantly lower compared to that in the control (3.05 μg per bead, p < 0.05), blank micelles (3.12 μg per bead, p < 0.05), HK-M nano-micelles (1.53 μg per bead, p < 0.05), and DOX-M nano-micelles (1.77 μg per bead, p < 0.05) groups. These results indicated that DOX-HK-M nano-micelles significantly suppressed tumor angiogenesis in tumor-bearing mice, which might participate in the inhibition of tumor growth and metastasis.

### Determination of tumor cell proliferation *in vivo*

**Figure 16:**
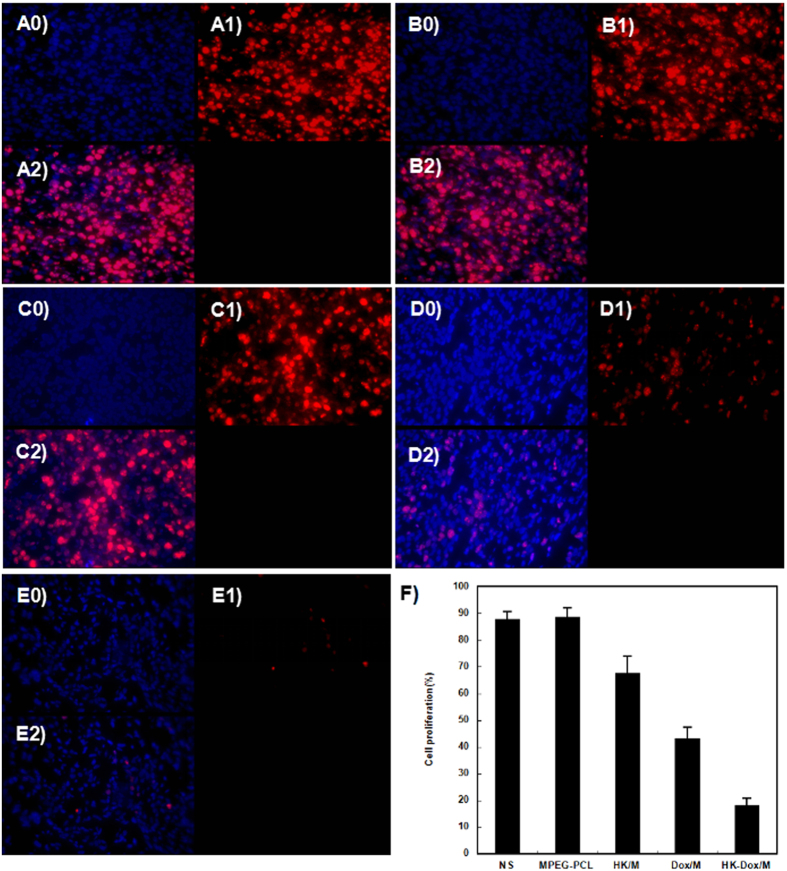
Ki-67 immunohistochemical analysis. Tumor tissue was collected after the final treatment and tumor sections from the groups treated with intravenous saline (**A**), empty MPEG-PCL micelles (**B**), HK-M (**C**), Dox-M (**D**), or Dox-HK-M (**E**) were subjected to immunohistochemistry to detect Ki-67, a marker of proliferation. (**F**) The percent of apoptotic cells in each group (Mean ± SEM, n = 5). The Dox-HK-M micelles inhibited ovarian cancer growth more effectively than other group.

Immunohistochemical staining of Ki-67 was employed to examine the proliferative potential of tumor cells. In Fig. 16A–E, tumor tissue in the DOX-HK-M nano-micelles group showed fewer Ki-67 positive cells than that of other groups under a similar high–power field. The number of Ki-67 in the DOX-HK-M nano-micelles group (18.3%) was significantly lower compared with that of the control (88.1%, p < 0.05), blank micelles (88.7%, p < 0.05), HK-M nano-micelles (67.7%, p < 0.05), and DOX-M nano-micelles (43.3%, p < 0.05) groups, respectively.

## Discussion

Chemotherapy is one of the most basic treatment strategies for cancer. Unfortunately, the administration of anti-cancer drugs often causes serious side effects such as nausea, vomiting, diarrhea, and hair loss, which limits the dose and frequency of chemotherapy. In recent years, nanomedicine, with fewer side effects and well-controlled release of drugs, has shown promising applications in the field of drug delivery^27^,^28^. DOX is a hydrophobic drug used mainly for the treatment of lung, breast, liver, prostate, and brain cancers, with the cardiac toxicity limiting the dose that can be used^29^,^30^. HK, on the other hand, was demonstrated to be a potential chemosensitizer and have therapeutic effect in various cancers^13^,^31^,^32^, but the therapeutic effect of HK is restricted by its poor water solubility.

In this study, we used MPEG-PCL micelles to co-deliver HK and DOX, and successfully developed an intravenously-injectable formulation. Furthermore, our formulation was characterized in detail, and its anti-angiogenic and anti-tumor effects were evaluated both *in vitro* and *in vivo*. In this protocol, the DOX-HK-M nano-micelles were prepared by a two-step nano-precipitation method, which was easy to scale up. The MPEG-PCL copolymer self-assembled into core-shell structures, encapsulating HK, DOX, or DOX-HK during the process. The DL and EL of the DOX-HK-M nano-micelles were 5% and 93.4%, respectively. The DOX-HK-M nano-micelles were monodisperse, with an average particle size of about 34 nm. As confirmed by the transparent appearance of DOX-HK-M nano-micelles, encapsulation in the nano-micelles enabled both DOX and HK to completely disperse in aqueous solution (Fig. 2D).

Several investigations about the therapeutic effects of the DOX-HK-M nano-micelles were made in this work. An *in vitro* release study showed that DOX-HK-M nano-micelles released DOX and HK more slowly than that of HK-M nano-micelles or DOX-M nano-micelles individually. Combining their small size (34 nm) and slow release of DOX and HK, the DOX-HK-M nano-micelles could potentially circulate *in vivo* for a long time after systemic application, which may help to maintain plasma drug concentrations.

Our DOX-HK-M nano-micelles showed stronger anti-cancer effects than DOX-M nano-micelles or HK-M nano-micelles both *in vitro* and *in vivo. In vitro*, DOX-HK-M nano-micelles caused increased cytotoxicity and induction of apoptosis in C6 glioma cells when compared with DOX-M or HK-M nano-micelles, and no difference was observed between the blank micelles-treated and control group, as confirmed by an MTT assay, Hoescht staining, and FCM analysis. Moreover, *in vivo* studies showed that systemic application of DOX-HK-M nano-micelles was more efficient in inhibiting tumor growth than DOX-M or HK-M nano-micelles in a subcutaneous C6 glioma model, which was further confirmed by the staining of TUNEL, CD31, and Ki67 in tumor tissues. In addition, the anti-tumor effects were also investigated in a tumor xenograft zebrafish model, which indicated stronger inhibition of tumor growth in the DOX-HK-M nano-micelles treated group than that of other groups. Finally, a cellular uptake study was implemented by using a fluorescence microscope, with its results suggesting that the increased cytotoxicity of DOX-HK-M nano-micelles was related to their enhanced cellular uptake.

Zebrafish are widely used as an ideal model to screen small molecules that affect vascular formation due to their well-described and highly conserved vascular system^33^,^34^. In this work, Tg (FLK-1: EGFP) zebrafish were used to investigate the inhibitory effects of DOX-HK-M nano-micelles on embryonic angiogenesis, as their endothelial cells exhibit green fluorescence. In the embryonic anti-angiogenesis assay, DOX-HK-M nano-micelles caused stronger inhibitory effects on angiogenesis than that of DOX-M nano-micelles or HK-M nano-micelles, while the blank micelles showed no inhibitory effects at all. In addition, the alginate-encapsulated tumor assay, a specific tumor angiogenesis model *in vivo*, demonstrated that DOX-HK-M nano-micelles could more efficiently inhibit tumor angiogenesis than that of DOX-M or HK-M nano-micelles. Therefore, the enhanced anti-tumor effects of DOX-HK-M nano-micelles may be due to direct cytotoxicity of cancer cells and inhibitory effects on tumor angiogenesis^35^,^36^,^37^,^38^,^39^.

Alternatively, angiogenic blood vessels in tumor tissues, which are different from those in normal tissues, have gaps between adjacent endothelial cells^40^. This defective structure, coupled with poorly developed lymphatic drainage, creates the enhanced permeability and retention (EPR) effect, which permits the nanoparticles to extravasate through these gaps into extravascular spaces and then accumulate inside tumor tissues^41^. For such a passive targeting mechanism (i.e., the EPR effect) to work, the circulation time of nanoparticles must be long enough. To maximize the targeting effect and circulation time, the optimal nanoparticle diameter must be less than 100 nm, and a hydrophilic surface is needed to avoid clearance by macrophages^40^. The monodisperse DOX-HK-M nano-micelles are small enough <100 nm) for this purpose. Indeed, the prepared DOX-HK-M nano-micelles also have a core-shell structure, allowing the hydrophilic PEG to act as a brush-like protective shell. As such, the particles’ small size and hydrophilic shell may contribute to increasing the accumulation of DOX and HK in tumor tissues due to the EPR effect, thus enhancing the anti-cancer effects of DOX and HK.

In this work, we successfully prepared intravenously-injectable DOX-HK-M nano-micelles, and their enhanced anti-cancer effects when compared to DOX-M and HK-M nano-micelles was due to the direct cellular cytotoxicity, stronger inhibitory effects on angiogenesis, and slower release, causing the EPR effect. In summary, our findings indicate that the DOX-HK-M nano-micelles have potential application in the treatment of glioma.

## Conclusion

Monodisperse DOX-HK-M nano-micelles were prepared by a self-assembly method, which rendered DOX and HK completely dispersible in aqueous solution. They had promising effects on inhibiting the growth of C6 glioma cells, both *in vitro* and *in vivo*, by directly killing cancer cells, inducing apoptosis, decreasing the density of tumor microvasculature, inhibiting angiogenesis, and EPR effects. In conclusion, DOX-HK-M nano-micelles, an excellent intravenously-injectable formulation of DOX and HK, hold promising clinical application in cancer therapy in the future.

## Materials and Methods

### Materials

Polyethylene glycol methyl ether (MPEG, Mn = 2000, Fluka, USA), ε-caprolactone (ε-CL, Alfa Aesar, USA), stannous octoate (Sn(Oct)_2_, Sigma, USA), doxorubicin (DOX, Sigma), methyl thiazolyl tetrazolium (MTT, Sigma, USA), Dulbecco’s modified eagle medium (DMEM Gibco, USA), fetal bovine serum (FBS, Gibco, USA), alginate sodium (Sigma, USA), fluorescein isothiocyanate-dextran (FITC-dextran, Sigma, USA), Annexin V-FITC/PI Detection kit (keyGEN Biotech, China), tricaine (Sigma, USA), 1-phyenyl-2-thiourea (PTU, Sigma, USA), and methanol (HPLC grade, Fisher scientific, UK) were used without further purification. HK was purchased from Suzhou NeuPharma Co. All the materials used in this article were analytic reagent (AR) grade and used as received.

C6 glioma cells were maintained and grown in DMEM supplemented with 10% FBS in a 37 °C incubator with a humidified 5% CO_2_ atomosphere.

Tg(flk:EGFP) transgenic zebrafish were provided by Dr. Shuo Lin (UCLA, USA). Female BALB/c nude mice (6–8 weeks old) were purchased from the Laboratory Animal Center of Sichuan University (Chengdu, China). Animal experiments were performed according to the guidelines of the Animal Care and Use Committee of Sichuan University (Chengdu, Sichuan, China) and approved by the Animal Care and Use Committee of Sichuan University.

### Preparation of DOX-M, HK-M, and DOX-HK-M nano-micelles

DOX-HK-M nano-micelles were prepared in two steps by a self-assembly method. First, HK (5 mg) and MPEG-PCL diblock copolymer (90 mg) were co-dissolved in 2 ml of acetone in order to form an organic phase. The mixture was then added into water under moderate mechanical stirring. With the diffusion of acetone into water, the amphiphilic MPEG-PCL and HK self-assembled into core-shell structured HK/MPEG-PCL nano-micelles with core-encapsulated HK. At last the mixture was evaporated in a rotary evaporator at 55 °C under reduced pressure to remove acetone. Secondly, 1 mL of 10x phosphate-buffered saline (PBS: 0.1 M, pH 7.4) was added to HK/MPEG-PCL nano-micelles (8 ml) and mixed completely. Next, 1 mL doxorubicin solution (5 mg/ml) was dripped slowly into the mixture under continuous stirring by a mechanical stirrer. The DOX-HK-M nano-micelles were prepared and are shown in Fig. 1C. The resulting DOX-HK-M nano-micelle solution was filtered by a 200 nm syringe filter (Millex-LG, Millipore Co, Billerica, MA) to remove the insoluble drugs and bacteria. Finally, the filtered DOX-HK-M nano-micelles were lyophilized and stored at 4 °C for further application. The HK/MPEG-PCL, Dox/MPEG-PCL, and empty MPEG-PCL micelle formulations were prepared in the same way as the Dox-HK/MPEG-PCL micelles, but without HK or doxorubicin in the mixtures when appropriate.

### Characterization of DOX-HK-M nano-micelles

The particle size distribution and zeta potential of prepared DOX-HK-M nano-micelles were measured by dynamic light scattering (DLS) (Malver Nano-ZS 90; Malvern Instruments, Malvern, UK). The temperature was kept at 25 °C during the measuring process. All results are the mean of three independent test runs.

The morphological characteristics of the DOX-HK-M nano-micelles were observed under a transmission electron microscope (TEM) (H-6009IV; Hitachi, Tokyo, Japan). Micelles were diluted with distilled water and placed on a copper grid covered with nitrocellulose. Samples were negatively stained with phosphotungstic acid and dried at room temperature before observation.

The drug loading (DL) and encapsulation efficiencies (EE) of DOX-HK-M, HK-M, and DOX-M nano-micelles were determined using HPLC. Briefly, 10 mg of lyophilized DOX-HK-M, HK-M, and DOX-M nano-micelles were dissolved in 0.1 mL of methanol. The concentrations of HK and DOX were determined by high-performance liquid chromatography (HPLC, Waters Alliance 2695; Waters, Milford, MA). The solvent delivery system was equipped with a plus auto-sampler and a column heater. Detection was conducted on a Waters 2966 detector. Chromatographic separations were performed on a reversed-phase C 18 column (4.6 × 150 nm, 5 μm, Sunfire Analysis column), and the column temperature was kept at 28 °C. Methanol-water (70/30, v/v) was used at a flow rate of 1 mL/min as eluent. The EE and DL of each micelle variant was calculated according to equations (1) and (2):


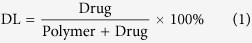



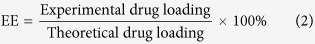


### Crystallographic studies

Crystallographic assays were performed on doxorubicin powder, honokiol powder, lyophilized blank MPEG-PCL nano-micelles, and DOX-HK-M nano-micelles using an X-ray diffractometer (XRD) (X’ Pert Pro, Philips, Netherlands) with Mo Kα radiation.

### Drug release *in vitro*

Modified dialysis methods were used to determine the *in vitro* release kinetics of DOX and HK from DOX-HK-M nano-micelles. Briefly, 0.5 mL of DOX-HK-M nano-micelle solution was placed in a dialysis bag (molecular weight cutoff, 3.5 kDa), and 0.5 mL of HK-M nano-micelles and DOX-M nano-micelles were used as controls. The dialysis tubes were incubated in 30 mL of PBS (pre-warmed to 37 °C, pH 7.4) containing 10% FBS at 37 °C with gentle shaking. The incubation media was replaced with fresh incubation media at predetermined time points. The drugs released into the incubation media were qualified using HPLC. The study was repeated three times, and results are expressed as mean value ± standard deviation (SD).

### Analysis of cytotoxicity

C6 glioma cells were maintained in a 37 °C incubator with a humidified 5% CO_2_ atmosphere. The cytotoxicity profiles of HK-M, DOX-M, and DOX-HK-M nano-micelles were evaluated by microtiter terazolium (MTT) assay. Cells in the exponential phase were seeded at densities of 5 × 10^3^ and 3 × 10^3^ cells per well in 96-well culture plates and grown for 24 h at 37 °C in the presence of 5% CO_2_. The cells were then exposed to each formulation at different concentrations ranging from 20 to 320 ng/mL for 24 or 48 hours, respectively. After these treatments, each well was added to 20 μL of 5 mg/mL MTT solution and then incubated for 3 hours. The supernatants were aspirated and 150 μL of dimethyl sulfoxide were added in each well to dissolve the formazan crystals for 10 minutes with shaking. Finally, the absorbance at 570 nm was read on a microplate reader. The mean percentage of cell survival relative to that of untreated cells was estimated using data from six individual experiments and all data are presented as the means ± SD. (The relative cell viability was estimated/determined by comparing the absorbance at 570 nm with the control wells which contained only cell culture media).

### Apoptosis assays

#### Hoescht staining assay

C6 glioma cells were treated by NS, blank micelles (0.08 μg/ml), HK-M nano-micelles (HK: 0.08 μg/mL), DOX-M nano-micelles (Dox: 0.08 μg/mL) and DOX-HK-M nano-micelles (HK and Dox: 0.08 and 0.08 μg/mL) for 48 hours in 24-well plates and washed with cold PBS twice. Hoescht 33342 was then added to the culture media at a final concentration of 5 μg/mL and the changes of nuclear morphology in treated cells were observed immediately under fluorescence microscopy with a filter for Hoescht 33342.

#### Flow cytometric analysis (FCM analysis)

After treatments with nano-micelles, an annexin V-FITC/PI (propidium iodide) apoptosis detection kit (BD PharMingen) was used to label the harvested C6 glioma cells according to the instruction of manufacture. A BD FACSCalibur (USA) flow cytometer was used to quantified the apoptotic and necrotic cells. The cells of early apoptotic (Annexin V-positive and PI-negative) or late apoptotic (Annexin V-positive and PI-positive) phases were included in cell death determinations.

### Cellular uptake study

For a qualitative study, the cellular uptake of blank micelles, H-M nano-micelles, DOX-M nano-micelles, and DOX-H-M nano-micelles was observed using a fluorescence microscope on C6 glioma cells. The cells were seeded at a density of 3 × 10^5^ cells per well in six-well plates. They were then incubated with blank micelles, H-M nano-micelles, DOX-M nano-micelles and DOX-H-M nano-micelles at a final DOX and/or HK concentration of 40 ng/mL and 80 ng/mL, respectively. Four hours after the incubation, the cells were washed with PBS thrice to remove excess micelles, and the cell nuclei were then stained with Hoescht. Fluorescence was observed using a fluorescence microscope. The treated C6 glioma cells were washed with PBS twice and the collected for FCM analysis. The FCM was used to analyze intracellular DOX fluorescence using 10,000 cells. Each experiment was performed in three independent replicates.

### Anti-angiogenesis activities in transgenic zebrafish model

An FLK-1 promoter EGFP transgenic (Tg (FLK-1: EGFP)) zebrafish line raised by standard methods as described in ref. 42 was used in an anti-angiogenic activity assay. The bright green and consistent fluorescence of blood vessels in Tg (FLK-1: EGFP) zebrafish embryos made the Tg (FLK-1: EGFP) zebrafish an idea model for investigating anti-angiogenic activity of drugs.

Embryos were maintained at 28 °C in Holtfreter’s solution after transplantation procedures. Embryos at 14 hours post-fertilization (hpf) were exposed to Holtfreter’s solution (control), blank micelles, H-M nano-micelles, DOX-M nano-micelles, and DOX-H-M nano-micelles at a final DOX and/or HK concentration of 1 μg/mL. At 24 hpf, embryos were stripped off the egg sheath and anaesthetized with 0.01% tricaine. Finally, the images of zebrafish blood vessels were taken under a Zeiss M2Bio fluorescence microscope (Carl Zeiss Microimaging Inc.). Ten embryos per group were used for these experiments, and each experiment was performed in three independent replicates.

### Anti-tumor activities in a transgenic zebrafish model

Our previous work showed that implanting U87 tumor cells into the perivitelline space of zebrafish allowed for tumor growth in zebrafish embryos^43^. Therefore, these zebrafish with tumor xenografts could serve as an ideal model to investigate the anti-tumor effect of our nano-micelles. Indeed, the U87 tumor cells were also transfected with EGFP by lentivirus in order to aid in analysis. Zebrafish embryos were stripped off the egg sheath and anesthetized with 0.01% tricaine at 48 hpf. Using a Zeiss Stemi 2000-C dissecting microscope (Carl Zeiss Microimaging Inc., Thornwood, NY), zebrafish were then injected with 300 tumor cells into the perivitelline space using a Cell Tram Vario injector (Eppendorf, USA) with a glass micropipette (50 mm length, diameter of the needle opening about 25 μg/mL). At 24 h post-implantation, Holtfreter’s solution (control), blank micelles, H-M nano-micelles, DOX-M nano-micelles, and DOX-H-M nano-micelles were added into the incubating Holtfreter’s solution at a final DOX and/or HK concentration of 1 μg/mL. Finally, images of the tumors were taken using a confocal microscope (DM6000 CS, Leica, Germany) at 5 days post-implantation.

### Subcutaneous injection of C6 glioma cells

A brain tumor model was established by subcutaneous injection of 1 × 10^7^ C6 glioma cells in exponential phase into the right flank of BALB/c nude mice (6–8 weeks, 20 ± 2 g). Mice bearing tumors around 100 mm^3^ were selected and randomly assigned to 5 groups (5 mice per group). The five groups were intravenously injected with NS (control), blank micelles (3 mg/kg), HK-M nano-micelles (3 mg/kg), DOX-M nano-micelles (3 mg/kg), or DOX-HK-M nano-micelles (3 mg/kg) four times, once every five days. The tumor-bearing mice in each group were weighed every three days. The tumor size was also measured externally every three days using calipers during the experimental period. The tumor volume was approximated according to the following equation: tumor volume = 0.52 × length × width^2^. The mice were sacrificed at the end of the experiment. Solid tumors from each group were then harvested and processed for subsequent immunohistochemical analysis and terminal deoxynuleotidyl transferase-mediated dUTP nick-end labeling (TUNEL) assay, described below.

### TUNEL assay

Cellular apoptosis in C6 gliomas was determined using a TUNEL assay. First, the subcutaneous tumor tissues described above from each group were harvested, fixed in 4% (w/v) paraformaldehyde, embedded in paraffin, and sectioned according to conventional methods. TUNEL staining was performed with an *in situ* cell death detection kit (DeadEnd^TM^ Fluorometric TUNEL System, Promega, Madison, USA) according to the manufacturer’s protocol. Five high-power fields of sections from each group were randomly selected and analyzed. The apoptotic index was calculated as the number of apoptotic cells in each high-power field.

### Alginate-encapsulated tumor cell assay

An alginate-encapsulated tumor cell assay was carried out as previously described to evaluate the anti-angiogenic activity of DOX-HK-M nano-micelles^44^. Briefly, C6 glioma cells were re-suspended in an alginate solution (1.5% w/v) and then dropped into a calcium chloride solution (250 mM) to form alginate beads containing 1 × 10^5^ tumor cells per bead. At day 0, the prepared alginate beads were subcutaneously implanted into an incision made on the dorsal sides of nude BALB/c mice. Afterwards, fifteen bead-bearing mice were randomly assigned to five groups (3 mice per group), and were intravenously injected with NS (control), blank micelles (3 mg/kg), HK-M nano-micelles (3 mg/kg), DOX-M nano-micelles (3 mg/kg), or DOX-HK-M nano-micelles (3 mg/kg) once a day for 3 days. Finally, mice were intravenously injected with 0.1 mL of a 100 mg kg-1 FITC-dextran solution at day 12. Alginate beads were imaged and rapidly removed within 20 minutes following the injection of FITC-dextran solution. The uptake of FITC-dextran was measured as described previously^44^.

### Immunohistochemical analysis

The angiogenic activity and cell proliferative potential of each tumor was detected by CD31 or Ki-67 immunofluorescence staining of paraffin-embedded tumor tissue sections, described above. Briefly, frozen sections of tumor tissue from each group were fixed in acetone, washed with PBS, and then stained with a rat anti-mouse CD31 polyclonal antibody (BD Pharmingen^TM^, USA) or rabbit anti-mouse Ki67 monoclonal antibody (Abcam, USA). Subsequently, the sections were washed with PBS twice, and were then incubated with a rhodamine-conjugated secondary antibody (Abcam, USA). The microvessel density (MVD) was determined by counting the number of microvessels per high-power field (400x magnification) in each section using a fluorescence microscope. Five high-power fields of tumor section were randomly selected and analyzed in a blind fashion by two independent investigators for this procedure. The Ki-67 labeling index (Ki-67 LI) was used to quantify the expression of Ki-67 by calculating the number of KI-67-positive cells per total number of cells.

### Statistical analysis

All data were expressed as the means ± SD. Statistical analysis was performed by one-way analysis of variance (ANOVA) (Tukey test) using SPSS software, version 11.5. Differences were considered to be statistically significant if p < 0.05.

## Additional Information

**How to cite this article**: Gao, X. *et al*. Enhancing the anti-glioma therapy of doxorubicin by honokiol with biodegradable self-assembling micelles through multiple evaluations. *Sci. Rep.* **7**, 43501; doi: 10.1038/srep43501 (2017).

**Publisher's note:** Springer Nature remains neutral with regard to jurisdictional claims in published maps and institutional affiliations.
